# Assessment of genetic integrity, splenic phagocytosis and cell death potential of (*Z*)-4-((1,5-dimethyl-3-oxo-2-phenyl-2,3dihydro-1*H*-pyrazol-4-yl) amino)-4-oxobut-2-enoic acid and its effect when combined with commercial chemotherapeutics

**DOI:** 10.1590/1678-4685-GMB-2017-0091

**Published:** 2018-02-19

**Authors:** Rodrigo Juliano Oliveira, Naiara da Cruz Leite Santos, João Renato Pesarini, Beatriz Carneiro de Oliveira, Claudia Rodrigues Berno, Flávio Henrique Souza de Araújo, Ingridhy Ostaciana Maia Freitas da Silveira, Raquel Oliveira Nascimento, Andréia Conceição Milan Brochado Antoniolli-Silva, Antônio Carlos Duenhas Monreal, Adilson Beatriz, Dênis Pires de Lima, Roberto da Silva Gomes

**Affiliations:** 1 Centro de Estudos em Células Tronco, Terapia Celular e Genética Toxicológica, Hospital Universitário “Maria Aparecida Pedrossian”, Empresa Brasileira de Serviços Hospitalares, Campo Grande, MS, Brazil; 2 Programa de Mestrado em Farmácia, Centro de Ciências Biológicas e da Saúde, Universidade Federal de Mato Grosso do Sul, Campo Grande, MS, Brazil.; 3 Programa de Pós-graduação em Saúde e Desenvolvimento na Região Centro-Oeste, Faculdade de Medicina “Dr. Hélio Mandetta”, Universidade Federal de Mato Grosso do Sul, Campo Grande, MS, Brazil.; 4 Programa de Pós-graduação em Química, Instituto de Química, Universidade Federal de Mato Grosso do Sul, Campo Grande, MS, Brazil.; 5 Laboratório de Síntese e Modificação Molecular, Faculdade de Ciências Exatas e Tecnologias, Universidade Federal da Grande Dourados, Dourados, MS, Brazil.

**Keywords:** Splenic phagocytosis, comet assay, micronucleus test, cell death, chemoprevention

## Abstract

The increased incidence of cancer and its high treatment costs have encouraged the search for new compounds to be used in adjuvant therapies for this disease. This study discloses the synthesis of (*Z*)-4-((1,5-dimethyl-3-oxo-2-phenyl-2,3dihydro-1*H*-pyrazol-4-yl) amino)-4-oxobut-2-enoic acid (IR-01) and evaluates not only the action of this compound on genetic integrity, increase in splenic phagocytosis and induction of cell death but also its effects in combination with the commercial chemotherapeutic agents doxorubicin, cisplatin and cyclophosphamide. IR-01 was designed and synthesized based on two multifunctionalyzed structural fragments: 4-aminoantipyrine, an active dipyrone metabolite, described as an antioxidant and anti-inflammatory agent; and the pharmacophore fragment 1,4-dioxo-2-butenyl, a cytotoxic agent. The results indicated that IR-01 is an effective chemoprotector because it can prevent clastogenic and/or aneugenic damage, has good potential to prevent genomic damage, can increase splenic phagocytosis and lymphocyte frequency and induces cell death. However, its use as an adjuvant in combination with chemotherapy is discouraged since IR-01 interferes in the effectiveness of the tested chemotherapeutic agents. This is a pioneer study as it demonstrates the chemopreventive effects of IR-01, which may be associated with the higher antioxidant activity of the precursor structure of 4-aminoantipyrine over the effects of the 1,4-dioxo-2-butenyl fragment.

## Introduction

Cancer comprises a group of diseases characterized by the progressive accumulation of mutations in the genome of a cell. These mutations lead to the altered expression or function of genes important for the maintenance of homeostasis, causing the loss of cell proliferation control (Steward and Brown, 2013). The genesis of cancer can occur via mutations (Ames *et al.*, 1973); therefore, the chemopreventive and chemotherapeutic potential of synthetic compounds that are able to reduce or increase the frequency of DNA damage has been explored, yielding good models for genetic toxicology (de Araújo *et al.*, 2017).

Nearly 2000 natural and synthetic compounds, including anti-inflammatory and antioxidant chemicals, have shown chemoprotective activity in preclinical trials, with good results also achieved in chemoprevention studies (Kim *et al.*, 2002).

The class of pyrazolones and its derivatives, such as antipyrines, aminoantipyrines and dipyrones, comprises compounds with antioxidant activity (Pisoschi and Pop, 2015). This group also includes 4-aminoantipyrine, one of the active metabolites of dipyrone (Hedenmaln and Spigset, 2002), an anti-inflammatory, antipyretic and analgesic nonsteroidal drug (Salgado *et al.*, 2015).

In another line of research involving the development of anticancer drugs, the pharmacophore fragment 1,4-dioxo-2-butenyl stands out because of its cytotoxic activity and ability to reduce cell proliferation (Jha *et al.*, 2010). These are desirable characteristics in chemotherapeutic agents because they can be associated with good regulators of the cell cycle and cause the elimination of cells with DNA damage, such as tumor cells.

The possibility of success is enhanced by the ability of these structural fragments to interfere in early stages of carcinogenesis, acting on molecular and/or cellular targets specific to inflammatory and proliferation processes (Pathak *et al.*, 2003).

To produce a compound that would have all the above characteristics, we conducted the synthesis of (*Z*)-4-((1,5-dimethyl-3-oxo-2-phenyl-2,3dihydro-1*H*-pyrazol-4-yl) amino)-4-oxobut-2-enoic acid (IR-01), which has the pharmacophore group 1,4-dioxy-2-butenyl as its structure base and contains the fragment 4-aminoantipyrine (an active dipyrone metabolite). Our aim was to develop a molecule with specific and effective therapeutic applications in the prevention and/or treatment of cancer.

In addition to the biological properties, the synthetic design took into account the low cytotoxicity of *N*-aryl-maleamic acids, which is attributable to the interaction between the two carboxyl groups and the olefinic fragment that may hinder the passage of the compounds through the cell membrane. The hypothesis proposed in the literature (Jha *et al.*, 2010) proposes that the cytotoxic capacity of the compounds containing these fragments is primarily controlled by the olefinic and aryl groups and the spatial arrangement between these fragments, which can directly affect the compound’s access through the lipid bilayer to the interior of the cell. The polarity balance may facilitate both the passage of the compound through the cell membrane (which would potentiate its biological effects) and the excretion of the compound from the body (after exerting its biological effects).

This study reports the synthesis of IR-01, taking into consideration the structural characteristics described above and the evaluation of IR-01 regarding genetic integrity, splenic phagocytosis evaluation and the induction of cell death. Furthermore, the study describes the effects of IR-01 in combination with the commercial chemotherapeutic agents doxorubicin, cisplatin and cyclophosphamide.

## Material and Methods

### Chemistry

#### Synthesis of IR-01

**Figure 1:**
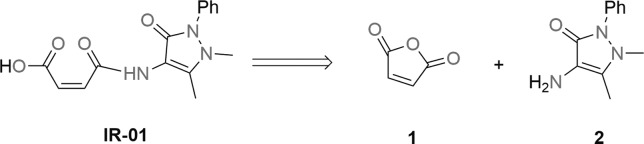
Retrosynthetic analysis for (*Z*)-4-((1,5-dimethyl-3-oxo-2-phenyl-2,3dihydro-1*H*-pyrazol-4-yl) amino)-4-oxobut-2-enoic acid.

Starting with low-cost materials and a one-pot procedure, the reaction of maleic anhydride with the corresponding amine was performed using a microwave reactor but no solvent, for a cleaner methodology with high reproducibility and good yield, further lending validity to the method (Figure 1).

**Figure 2:**
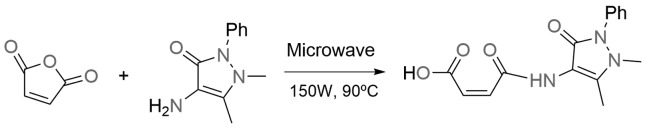
Synthesis of (*Z*)-4-((1,5-dimethyl-3-oxo-2-phenyl-2,3dihydro-1*H*-pyrazol-4-yl) amino)-4-oxobut-2-enoic acid.

The formation of the synthetic target occurs after the attack of the 4-aminoantipyrine nitrogen on the carbonyl carbon of maleic anhydride, which provides ring opening and the subsequent formation of the acid of interest with good yield (Figure 2).

#### Reagents and techniques

All reagents and spectrograde solvents for synthesis and NMR measurements were purchased commercially and used without further purification.

The melting point was determined on a Quimis dry melting point apparatus, model Q340S23, and used as uncorrected data. The microwave procedure was performed in a CEM/Discover microwave reactor with sealed tube.

^1^H and ^13^C NMR spectra were recorded at room temperature on a Bruker 300 spectrometer (10% in deuterated dimethylsulfoxide (DMSO-d_6_) solutions at 298 K) operating at 300.132 and 75.476 MHz, respectively. Data processing was conducted on a Solaris workstation. The ^1^H and ^13^C chemical shifts are reported on the δ scale (ppm) and referenced to internal DMSO-d_6_; coupling constants *J* are reported in hertz (Hz). The abbreviations s, d and m represent simplet, douplet and multiplet, respectively.

#### Synthesis process

In a sealed tube, 4-aminoantipyrine (2.0 g, 10 mmol) and maleic anhydride (1.0 g, 10 mmol) were subjected to microwave irradiation (150 W) at 90 °C for 10 s. The solid was washed with ethyl acetate and filtered. The remaining yellow solid was recrystallized from CH_3_Cl, giving IR-01 (2.79 g, 93%). ^1^H NMR (DMSO-d_6_, 300 MHz) δ (ppm): 2.13 (s, 3H), 3.02 (s, 3H), 6.26 (d, 1H, *J*_*cis*_ = 12.3 Hz), 6.48 (d, 1H, *J*_*cis*_ = 12.3 Hz), 7.30 (m, 3H), 7.46 (m, 2H). ^13^C NMR (CDCl_3_, 75 MHz) δ (ppm): 11.71 (CH_3_), 36.24 (CH_3_), 106.62 (C), 124.22 (CH), 126.94 (CH), 129.60 (CH), 131.54 (CH), 131.60 (CH), 135.25 (C), 152.56 (C), 161.73 (C=O), 164.58 (C=O), 167.03 (C=O). Melting point: 178.1-179.8 °C.

### Chemical agents, animals and experimental design

The DNA-damage-inducing agents (commercial chemotherapeutic agents) used in this study were the following: doxorubicin (Glenmark Pharmaceuticals Ltd., Argentina. MS Reg. No. 1.1013.0232.002-4, Lot #21130040) at a dose of 16 mg/kg body weight (b.w.) intraperitoneally (*ip*) cisplatin (Accord Pharmaceuticals Ltd., UK. MS Reg. No. 1.5537.0002.003-7, Lot #88549) at a dose of 6 mg/kg (b.w., *ip*), and cyclophosphamide (Genuxal^®^, Baxter Ltda., Germany. MS Reg. No. 1.00683.0168.003-1, Lot #F728) at a dose of 100 mg/kg (b.w., *ip*). Doxorubicin and cyclophosphamide were diluted in distilled water.

IR-01 was first diluted in 5% DMSO and then in glycated serum before the drug was administered at doses of 12, 24 and 48 mg/kg (b.w., *ip*).

Eighty Swiss female mice (with a mean weight of 30 g, 6-8 weeks old) were randomly distributed into 16 experimental groups (n = 5).

The animals were housed in individual cages lined with wood shavings on a ventilated rack (Alesco^®^) and provided commercial feed (Nuvital^®^) and filtered water *ad libitum*. The experimental conditions were controlled, with a 12-hour light:12-hour dark photoperiod, mean temperature of 22 2 °C and mean humidity of 55 ± 10%. The experiment was approved by the Animal Ethics Committee of the Federal University of Mato Grosso do Sul (Universidade Federal de Mato Grosso do Sul - UFMS) under protocol no. 399/2012 and performed according to the Declaration of Animal Rights.

For the evaluation of the IR-01 effects, the following experimental groups were established:

In lot 1, a negative control group comprised animals that received a dose of distilled water and another of 5% DMSO in glycated serum, both at 0.1 mL/10 g (b.w., *ip*). The IR-01 groups in lot 1 comprised animals treated with IR-01 at concentrations of 12, 24 and 48 mg/kg (b.w., *ip*) and with a dose of distilled water at 0.1 mL/10 g (b.w., *ip*).

To assess the effects of combining IR-01 with the commercial chemotherapeutic agents, the following experimental groups were established.

In lot 2, a doxorubicin group (DOX) comprised animals treated with doxorubicin at a dose of 16 mg/kg (b.w., *ip*) and with 5% DMSO in glycated serum at a dose of 0.1 mL/10 g (b.w., *ip*). The DOX + IR-01 groups in lot 2 comprised animals that were treated with doxorubicin at a dose of 16 mg/kg (b.w., *ip*) and IR-01 at doses of 12, 24 and 48 mg/kg (b.w., *ip*).

The animals in lots 3 and 4 were treated as described in lot 2 except that doxorubicin was replaced by cisplatin (CIS and CIS + IR-01) and cyclophosphamide (CPP and CPP + IR-01) at doses of 6 and 100 mg/kg (b.w., *ip*), respectively.

At 24 (T1), 48 (T2) and 72 (T3) hours after the treatments, 20 μL of peripheral blood was collected to perform a micronucleus assay. Additionally, 20 μL of peripheral blood was collected at T1 to perform a comet assay. A new aliquot was collected at T3 for a differential blood cell count. At the end of the experiment, at T3, the animals were euthanized by cervical dislocation to collect the spleen for a phagocytosis test and the kidney and liver for cell death analysis.

### Biological assays

#### Peripheral blood Comet assay

The comet assay was performed according to the protocol of Singh *et al.* (1988), with modifications by Oliveira *et al.* (2015a). The material was analyzed using an epifluorescence microscope (Bioval^®^, model L 2000A) with a 40 objective, a 420-490 nm excitation filter and a 520 nm barrier filter. As described by Kobayashi *et al.* (1995), a total of 100 cells per treatment were inspected visually and the comets were classified as: class 0, undamaged cells showing no tail; class 1, cells with a tail size smaller than the diameter of the nucleoid; class 2, cells with a tail size 1- 2 times the diameter of the nucleoid; class 3, cells with a tail size greater than two times the diameter of the nucleoid. Apoptotic cells that showed a totally fragmented nucleus were not scored. The total score was calculated as the sum of the number of cells scored for each class times that class value.

#### Peripheral blood Micronucleus assay

The micronucleus assay in peripheral blood was performed according to Hayashi *et al.* (1990), with modifications by Oliveira *et al.* (2015a). A 20 μL peripheral blood aliquot was covered with a cover slip after its deposition on a slide precoated with 20 μL of acridine orange (1.0 mg/mL). The slide was stored in a freezer (-20 °C) for at least seven days. The analysis was performed under an epifluorescence microscope with a 40 objective (Bioval^®^, model L 2000A) along with a 420-490 nm excitation filter and a 520 nm barrier filter. Two thousand cells were analyzed per animal.

#### Cell death assay

One hundred microliters of a macerated liver or kidney solution was placed on a slide. Next, the slide was fixed in Carnoy’s solution for 5 min and was then subjected to a decreasing series of ethanol concentrations (95-25%), washed with McIlvaine’s buffer for 5 min, stained with 0.01% acridine orange for 5 min and washed again with buffer. Dying cells were identified through an analysis of the DNA fragmentation patterns, according to Carvalho *et al.* (2015) and Navarro *et al.* (2014).

#### Splenic phagocytosis assay

The spleen was macerated in saline solution. One hundred microliters of cell suspension was covered with a coverslip after its placement on a slide previously coated with 20 μL of acridine orange (1.0 mg/mL). The slides were stored in a freezer until their analysis, which was performed with a fluorescence microscope (Bioval^®^, model L 2000A) using a 40 objective along with a 420-490 nm filter and a 520 nm barrier filter. Two hundred cells were analyzed per animal. The presence or absence of phagocytosis was determined based on the descriptions of Carvalho *et al.* (2015) and Hayashi *et al.* (1990).

#### Differential blood cell count

A 20 μL aliquot of peripheral blood was used to prepare blood smears on glass slides. These slides were air dried and stained with a panoptic kit for 10 min. The cells were visualized under bright field microscopy using a 100 objective. A total of 100 cells per animal were analyzed and classified as lymphocytes, neutrophils, monocytes, eosinophils and basophils (Ishii *et al.*, 2011).

#### Calculation of percent damage reduction (%DR) and percent damage increase (%DI)

Manoharan and Banerjee (1985) and Waters *et al.* (1990) proposed the calculation of percent damage reduction to assess the chemopreventive ability of a substance when it is associated with a substance known to be mutagenic, such as the commercial chemotherapeutic agents used as positive control (PC). According to Oliveira *et al.* (2015a) and Navarro *et al.* (2014), the same calculation can be used to estimate the increase in DNA damage. Thus, for the present study, both the percent DNA damage reduction and percent DNA damage increase were calculated using the same formula:

00DR or 00DI =(Mean of PC − (Mean of IR−01 + PC)Mean of PC − Mean of Control )× 100 

#### Statistical Analysis

Data are reported as the mean ± standard error of the mean (SEM) and analyzed using Student’s *t*-test or the Mann-Whitney test, depending on whether the data distribution was parametric or nonparametric, respectively, using GraphPad InStat Demo version 3.6 (GraphPad Software Inc., San Diego, CA, USA). The significance level adopted was *p* < 0.05.

## Results

### Synthesis

The product formed was characterized by ^1^H and ^13^C NMR, and the results described below demonstrated chemical shifts and integrations consistent with IR-01.

**Figure 3:**
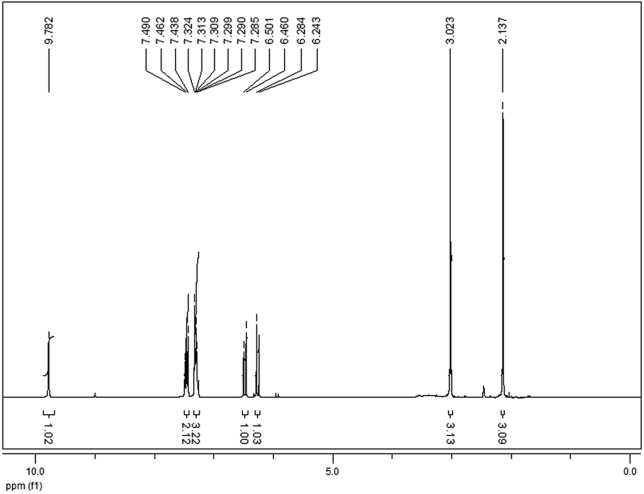
^1^H RMN spectra of IR-01 in DMSO-d_6_ at 300 Mhz.

In the ^1^H NMR spectra (Figure 3), two signals at the 6.26 and 6.48 ppm regions (J = 12.3 Hz), representing the two olefinic hydrogen doublets of the 1,4-dioxo-butenyl fragment, indicate the *Z* configuration of the compound obtained; the signal at 9.78 ppm refers to the amidic hydrogen.

**Figure 4:**
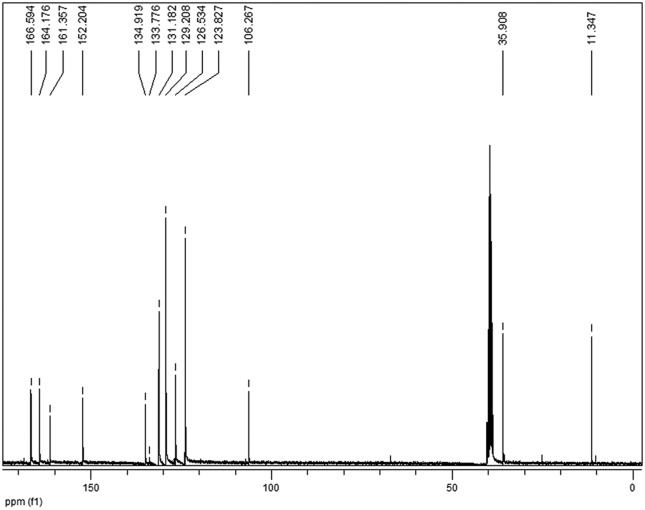
^13^C RMN spectra of IR-01 in DMSO-d_6_ at 75 MHz.

In the ^13^C NMR spectra (Figure 4), the three signals observed between 161.35 and 166.59 ppm, representing the IR-01 carbonyls, confirm the formation of the synthetic target.

All other ^1^H and ^13^C NMR signals are in agreement with the data reported in the literature for the same compound (Cunha *et al.*, 2005).

### Biological assays

#### Assessment of genetic integrity and effects of IR-01 on DNA damage caused by commercial chemotherapeutic agents

The genetic integrity assessment indicated that IR-01 can cause genomic damage (comet assay) but is unable to cause chromosomal damage (micronucleus assay). The treatments with the test compound caused an increase (*p* < 0.05) in the frequency of DNA damage by 2.37 and 4.44 and in the score by 2.3 and 4.05 for the 24 and 48 mg/kg doses, respectively (Table 1). The micronucleus frequency ranged from 0.6 ± 0.24 to 3.0 ± 0.44 in the control group and from 3.0 ± 0.31 to 5.2 ± 0.66 in the IR-01-treated groups (Table 2).

When associated with the commercial chemotherapeutic agents, IR-01 showed chemopreventive activity for most associations, except for the two highest doses when administered with cisplatin and evaluated in the comet assay (Table 1) and the intermediate dose in combination with cyclophosphamide in the icronucleus assay (Table 2).

The percent damage reduction by IR-01 in the Comet assay ranged between 4.17 and 36.11% in combination with doxorubicin and between 18.87 and 94.83% in combination with cyclophosphamide and was 72.73% for the lowest IR-01 dose in combination with cisplatin. An increase in percent damage was observed for the two highest IR-01 doses in combination with cisplatin (172.73 and 263.64% for 24 and 48 mg/kg, respectively) and for the lowest IR-01 dose in combination with cyclophosphamide (25.86%) (Table 1).

In the micronucleus assay, the percent damage reduction ranged from 65.35 to 91.43% for the combination with doxorubicin, from 62.50 to 86.67% for the combination with cisplatin and from 0 to 14.60% for the cyclophosphamide combination. For the latter, an increase in DNA damage that reached 56.02% was observed at the intermediate dose (Table 2).

#### Evaluation of the splenic phagocytosis potential and effects of IR-01 in combination with commercial chemotherapeutic agents

When administered alone, the two highest doses of IR-01, 24 and 48 mg/kg, increased (*p* < 0.05) the rate of splenic phagocytosis by 1.43 and 1.67, respectively (Table 3).

**Table 1:** Results of the comet assay showing the ability of IR-01 to cause or prevent genomic damage.

Experimental groups	Damaged cells	Damage classes				Score	%DR
		0	1	2	3		
**LOT 1**							
NC	16 ± 1.77	81 ± 2.43	12 ± 1.43	2.6 ± 0.24	1.0 ± 0.31	20 ± 2.35^a^	-
IR-01 12 mg/kg	21 ± 1.71^a^	78 ± 1.71	15 ± 0.67	4.2 ± 0.86	2.0 ± 0.44	30 ± 3.39ª	-
IR-01 24 mg/kg	38 ± 0.86^a*^	61 ± 0.86	33 ± 0.96	3.8 ± 1.02	1.80.58	46 ± 2.95^a*^	-
IR-01 48 mg/kg	71 ± 0.37^a*^	28 ± 0.37	64 ± 1.56	5.4 ± 1.20	2.0 ± 0.83	81 ± 2.47^a*^	-
**LOT 2**							
DOX	88 ± 0.50^a^	11 ± 0.50	56 ± 1.12	18 ± 1.03	13 ± 0.67	134 ± 2.48^a^	-
+IR-01 12 mg/kg	62 ± 2.61^b^	37 ± 2.61	47 ± 1.24	13. ± 1.24	1.20.58	77 ± 4.30^b^	36.11
+IR-01 24 mg/kg	73 ± 2.47^b^	26 ± 2.47	49 ± 2.70	20 ± 1.64	4.6 ± 1.16	103 ± 4.83^b^	20.83
+IR-01 48 mg/kg	85 ± 0.86^b^	14 ± 0.86	55 ± 3.95	252.51	4.4 ± 1.63	120 ± 4.05^b^	4.17
**LOT 3**							
CIS	27 ± 1.37^a^	72 ± 1.31	22 ± 1.77	4.4 ± 0.40	0.4 ± 0.40	35 ± 3.16^a^	-
+IR-01 12 mg/kg	19 ± 1.24^c^	80 ± 1.24	19 ± 1.18	0.2 ± 0.20	0.0 ± 0.0	19 ± 1.32^c^	72.73
+IR-01 24 mg/kg	46 ± 4.77^c^	53 ± 4.77	33 ± 5.62	8.8 ± 1.39	2.2 ± 0.96	59 ± 3.57^c^	-172.73
+IR-01 48 mg/kg	56 ± 1.65^c^	43 ± 1.65	44 ± 1.67	10 ± 0.87	1.6 ± 0.40	70 ± 1.94^c^	-263.64
**LOT 4**							
CPP	74 ± 2.01^a^	252.01	64 ± 2.21	10 ± 0.87	0.0 ± 0.0	85 ± 2.09^a^	-
+IR-01 12 mg/kg	89 ± 0.92^d^	10 ± 0.92	73 ± 1.63	16 ± 1.67	0.0 ± 0.0	101 ± 3.53^d^	-25.86
+IR-01 24 mg/kg	19 ± 2.52^d^	80 ± 2.65	14 ± 1.88	3.0 ± 0.70	1.8 ± 0.73	26 ± 3.81^d^	94.83
+IR-01 48 mg/kg	63 ± 3.52^d^	3522.8	57 ± 2.80	7.2 ± 0.86	0.0 ± 0.0	72 ± 1.22^d^	18.97

Data is represented as mean values ± standard error of the mean. %DR: Percent damage reduction; NC: Negative control group; DOX: Doxorubicin group; CIS: Cisplatin group; CPP: Cyclophosphamide group. ^(a)^Statistically compared to the NC group; ^(b)^Statistically compared to the DOX group; ^(c)^Statistically compared to the CIS group; ^(d)^Statistically compared to the CPP group; ^*^statistically different (*p* < 0.05; Mann-Whitney test).

**Table 2:** Results of the micronucleus assay related to the ability of IR-01 to cause or prevent chromosomal damage

Experimental groups	Mean ± SE			%DR		
	*24 h*	*48 h*	*72 h*	*24 h*	*48 h*	*72 h*
**LOT 1**							
NC	3.0 ± 0.44	1.8 ± 0.20	0.6 ± 0.24	-	-	-
IR-01 12 mg/kg	3.8 ± 0.37^a^	3.0 ± 0.31ª^*^	4.2 ± 0.37ª^*^	-	-	-
IR-01 24 mg/kg	4.2 ± 0.20^a^	4.6 ± 0.60ª^*^	4.8 ± 0.37ª^*^	-	-	-
IR-01 48 mg/kg	4.8 ± 0.37ª^*^	3.4 ± 0.50ª^*^	5.2 ± 0.66ª^*^	-	-	-
**LOT 2**							
DOX	52 ± 2.56^a*^	36 ± 1.72^a*^	26 ± 1.16ª^*^	-	-	-
+ IR-01 12 mg/kg	7.20.58^b*^	5.2 ± 0.37^b*^	7.2 ± 0.86^b*^	91.43	90.06	74.01
+ IR-01 24 mg/kg	12 ± 0.70^b*^	6.4 ± 0.50^b*^	7.2 ± 0.37^b*^	81.63	86.55	74.01
+ IR-01 48 mg/kg	13 ± 1.06^b*^	9.4 ± 0.40^b*^	9.4 ± 0.60^b*^	79.59	77.78	65.35
**LOT 3**							
CIS	27 ± 0.50ª^*^	20 ± 0.55^a*^	15 ± 0.50ª^*^	-	-	-
+ IR-01 12 mg/kg	6.2 ± 0.37^c*^	5.6 ± 0.24^c*^	4.2 ± 0.20^c*^	86.67	79.12	75.00
+ IR-01 24 mg/kg	7.0 ± 0.37^c*^	7.6 ± 0.50^c*^	6 ± 0.31^c*^	83.33	68.14	62.50
+ IR-01 48 mg/kg	8.4 ± 0.50^c*^	7.4 ± 0.60^c*^	5.8 ± 0.37^c*^	77.50	69.23	63.89
**LOT 4**							
CPP	41 ± 2.17^a*^	50 ± 2.34^a*^	28 ± 2.80^a*^	-	-	-
+ IR-01 12 mg/kg	33 ± 2.71^d^	63 ± 3.75^d^	24 ± 2.16^d^	21.05	-26.97	14.60
+ IR-01 24 mg/kg	55 ± 3.63^d*^	77 ± 2.95^d*^	42 ± 3.88^d*^	-36.84	-56.02	-51.10
+ IR-01 48 mg/kg	42 ± 3.85^d^	71 ± 2.70^d^	28 ± 2.10^d^	-2.63	-43.57	00.00

SE: Standard error of the mean; %DR: Percent damage reduction; NC: Negative control group; DOX: Doxorubicin group; CIS: Cisplatin group; CPP: Cyclophosphamide group. ^(a)^Statistically compared to the NC group; ^(b)^Statistically compared to the DOX group; ^(c)^Statistically compared to the CIS group; ^(d)^Statistically compared to the CPP group; ^*^statistically different (*p* < 0.05; Mann-Whitney test).

**Table 3:** Results related to splenic phagocytosis evaluation.

Experimental groups	Phagocytosis	
	Absolute values	Mean ± SE
**LOT 1**		
NC	221	44.2 ± 0.66
IR-01 12 mg/kg	228	45.6 ± 1.77ª
IR-01 24 mg/kg	316	63.2 ± 1.28ª^*^
IR-01 48 mg/kg	369	73.8 ± 1.15ª^*^
**LOT 2**		
DOX	670	134.0 ± 1.37ª^*^
+IR-01 12 mg/kg	321	64.2 ± 1.59^b*^
+IR-01 24 mg/kg	405	81.0 ± 1.30^b*^
+IR-01 48 mg/kg	373	74.6 ± 1.43^b*^
**LOT 3**		
CIS	269	53.8 ± 1.35ª^*^
+IR-01 12 mg/kg	106	21.2 ± 1.35^c*^
+IR-01 24 mg/kg	74	14.8 ± 1.02^c*^
+IR-0148 mg/kg	54	10.8 ± 0.86^c*^
**LOT 4**		
CPP	515	103.0 ± 2.00ª^*^
+IR-01 12 mg/kg	275	55.0 ± 3.46^d*^
+IR-01 24 mg/kg	316	63.2 ± 2.57^d*^
+IR-01 48 mg/kg	534	106.8 ± 3.13^d^

SE: Standard error of the mean; NC: Negative control group; DOX: Doxorubicin group; CIS: Cisplatin group; CPP: Cyclophosphamide group. ^(a)^Statistically compared to the NC group; ^(b)^Statistically compared to the DOX group; ^(c)^Statistically compared to the CIS group; ^(d)^Statistically compared to the CPP group; ^*^statistically different (*p* < 0.05; Student’s *t*-test).

In combination with doxorubicin, IR-01 reduced (*p* < 0.05) the frequency of phagocytosis by 47.91, 60.45 and 55.67% at the 12, 24 and 48 mg/kg doses, respectively. In combination with cisplatin, the respective reductions were 39.40, 27.51 and 20.07%. In combination with cyclophosphamide, the reductions were 53.40 and 61.36% for the 12 and 24 mg/kg doses, respectively (Table 3).

The differential blood cell count showed that at the three doses tested, IR-01 administered alone can increase (*p* < 0.05) the frequency of lymphocytes and reduce (*p* < 0.05) neutrophil and monocyte counts. Additionally, in the treatment with the commercial chemotherapeutic agents, an increase (*p* < 0.05) in the frequency of lymphocytes and a reduction in that of monocytes occurred for doxorubicin, cisplatin and cyclophosphamide, with a reduction (*p* < 0.05) in neutrophils also occurring for doxorubicin (Table 4).

The following results were observed for the combinations of chemotherapeutic agents with IR-01: (I) for doxorubicin, an increase (*p* < 0.05) in the frequency of lymphocytes and a reduction (*p* < 0.05) in that of monocytes for all the doses tested and a reduction (p < 0.05) in neutrophil frequency for the lowest and highest doses; (II) for cisplatin, an increase (*p* < 0.05) in the frequency of lymphocytes for the two higher doses and a reduction (*p* < 0.05) in that of neutrophils for all the doses; and (III) for cyclophosphamide, no statistically significant change (Table 4).

**Table 4:** Reference values and results related to the differential blood cell count.

Experimental Groups	Cell types				
	55-95%	10-40%	0.0-0.3%	0.0-0.4%	0.1-3.5%
	Lymphocytes^2^	Neutrophils^2^	Basophils^1^	Eosinophils^1^	Monocytes^2^
**LOT 1**					
NC	67 ± 0.74	22.0 ± 0.81	0 ± 0.001	0 ± 0.00	10.0 ± 0.40
IR-01 12 mg/kg	78 ± 1.48^a*^	11.0 ± 0.67^a*^	0 ± 0.00^a^	0 ± 0.00^a^	9.6 ± 0.50^a*^
IR-01 24 mg/kg	82 ± 1.00^a*^	8.0 ± 0.63ª^*^	0 ± 0.00^a^	0 ± 0.00^a^	6.8 ± 1.31^a*^
IR-01 48 mg/kg	86 ± 0.81^a*^	9.0 ± 0.87^a*^	0 ± 0.00^a^	0 ± 0.00^a^	4.6 ± 1.74^a*^
**LOT 2**					
DOX	76 ± 2.46ª^*^	12 ± 0.67ª*	0 ± 0.00^a^	2.81.31^a^	9.4 ± 1.83^a*^
+IR-01 12 mg/kg	85 ± 1.53^b*^	8.4 ± 1.03^b*^	0 ± 0.00^b^	1.80.58^b^	3.6 ± 0.92^b*^
+IR-01 24 mg/kg	85 ± 1.68^b*^	11.00.58^b^	0 ± 0.00 ^b^	1.4 ± 0.92^b^	1.4 ± 0.50^b*^
+IR-01 48 mg/kg	891.58^b*^	7.0 ± 0.47^b*^	0 ± 0.00^b^	2.5 ± 1.50^b^	1.2 ± 0.62^b*^
**LOT 3**					
CIS	78 ± 0.73ª^*^	17 ± 1.88^a^	0 ± 0.00^a^	0 ± 0.00^a^	3.4 ± 1.32^a*^
+IR-01 12 mg/kg	83 ± 2.62^c^	10 ± 1.20^c*^	0 ± 0.00^c^	0.2 ± 0.20^c^	6.4 ± 0.00^c^
+IR-01 24 mg/kg	85 ± 1.36^c*^	10 ± 0.96^c*^	0 ± 0.00^c^	0 ± 0.00^c^	4.2 ± 0.76^c^
+IR-01 48 mg/kg	85 ± 1.00^c*^	10 ± 0.50^c*^	0 ± 0.00^c^	0 ± 0.00^c^	4.4 ± 0.67^c^
**LOT 4**					
CPP	85 ± 3.49ª^*^	11 ± 3.53^a^	0 ± 0.00ª	0 ± 0.00^a^	2.81.06^a*^
+IR-01 12 mg/kg	85 ± 3.92^d^	10 ± 2.64^d^	0 ± 0.00^d^	0 ± 0.00^d^	4.2 ± 1.35^d^
+IR-01 24 mg/kg	92 ± 2.31^d^	4.6 ± 1.60^d^	0 ± 0.00^d^	0 ± 0.00^d^	3.0 ± 1.00^d^
+IR-01 48 mg/kg	86 ± 0.48^d^	11 ± 0.50^d^	0 ± 0.00^d^	0 ± 0.00^d^	1.8 ± 0.86^d^

Data is represented as mean values ± standard error of the mean. Statistical tests: ^(1)^Student’s *t*-test(p < 0.05) and ^(2)^Mann-Whitney test (*p* < 0.05). NC: Negative control group; ^(a)^Statistically compared to the NC group; ^(b)^Statistically compared to the DOX group; ^(c)^Statistically compared to the CIS group; ^(d)^Statistically compared to the CPP group; ^*^statistically different.

Neutropenia was observed in the groups treated with the two highest doses of IR-01, in DOX + IR-01 at the lowest and highest doses and in CPP + IR-01 at the intermediate dose. Eosinophilia was observed in the animals treated with cisplatin when combined with all doses of IR-01, and monocytosis occurred in the control groups treated with IR-01, in DOX, in DOX + IR-01 at the lowest dose, in CIS + IR-01 at all three doses, and in CPP + IR-01 at the lowest dose tested (Table 4).

#### Evaluation of cell death induction and effects of IR-01 in combination with commercial chemotherapeutic agents

The administration of IR-01 increased (*p* < 0.05) the frequency of dead cells in the liver by 1.69, 2.44 and 3.17 and in the kidneys by 2.05, 3.11 and 3.89 at the 12, 24 and 48 mg/kg doses, respectively (Table 5).

The commercial chemotherapeutic agents doxorubicin, cisplatin and cyclophosphamide caused an increase in dead cells frequency of 5.67, 7.07 and 6.98 in the liver and 2.79, 8.38 and 9.33 in the kidneys, respectively (Table 5).

The following results were observed for the chemotherapeutic agents tested in combination with IR-01: (I) for doxorubicin, the potentiation of cell death (p < 0.05) by 155.31% in the liver and 203.41% in the kidneys for the lowest IR-01 dose tested; (II) for cisplatin, a reduction of dead cells (*p* < 0.05) by up to 22.99% in the liver and 37.52% in the kidneys for the highest IR-01 dose; and (III) for cyclophosphamide, reductions of 32.29 and 64.44% in the liver and kidneys, respectively, also for the highest IR-01 dose (Table 5).

**Table 5:** Cell death evaluation on mice kidneys and liver.

	Liver		Kidneys	
Experimental Groups	Number of dead cells	Mean ± SE	Number of dead cells	Mean ± SE
**LOT 1**				
NC	88	17.6 ± 1.03	63	12.6 ± 0.81
IR-01 12 mg/kg	149	29.8 ± 1.93^a*^	129	25.8 ± 1.24^a*^
IR-01 24 mg/kg	215	43.0 ± 1.93^a^	196	39.20.58^a*^
IR-01 48 mg/kg	279	55.8 ± 0.86^a*^	245	49.0 ± 1.04^a*^
**LOT 2**				
DOX	499	99.8 ± 2.55^a*^	176	35.2 ± 2.57^a*^
+IR-01 12 mg/kg	779	1552.57^b*^	358	71.6 ± 3.40^b*^
+IR-01 24 mg/kg	610	122 ± 1.84^b*^	291	58.2 ± 1.39^b*^
+IR-01 48 mg/kg	565	113 ± 2.12^b*^	246	49.2 ± 2.28^b*^
**LOT 3**				
CIS	622	124.4 ± 6.03^a*^	528	1051.72^a*^
+IR-01 12 mg/kg	402	80.4 ± 5.92^c*^	297	59.4 ± 1.03^c*^
+IR-01 24 mg/kg	247	49.4 ± 3.80^c*^	221	44.2 ± 2.08^c*^
+IR-01 48 mg/kg	143	28.6 ± 2.42^c*^	197	39.4 ± 2.92^c*^
**LOT 4**				
CPP	614	122 ± 3.36^a*^	588	117 ± 1.16^a*^
+IR-01 12 mg/kg	307	61.4 ± 3.95^d*^	522	104 ± 1.93^d*^
+IR-01 24 mg/kg	362	72.4 ± 4.63^d*^	381	76.2 ± 3.35^d*^
+IR-01 48 mg/kg	197	39.4 ± 5.47^d*^	377	75.4 ± 2.08^d*^

SE: Standard error of the mean; NC: Negative control group; DOX: Doxorubicin group; CIS: Cisplatin group; CPP: Cyclophosphamide group. ^(a)^Statistically compared to the NC group; ^(b)^Statistically compared to the DOX group; ^(c)^Statistically compared to the CIS group; ^(d)^Statistically compared to the CPP group; ^*^statistically different (*p* < 0.05; Student’s *t*-test).

## Discussion

The increase in cancer incidence and the high cost of treatments motivate the search for new strategies to prevent and manage this disease (Mauro *et al.*, 2011). An approach with great potential is chemoprevention, which involves the use of natural and/or synthetic agents to suppress, inhibit or reverse the process of carcinogenesis in its early stages (Friedman and Rasooly, 2013).

Organic synthesis has gained prominence in the search to develop more potent and less toxic molecules, and the redesign and structural modification of previously known compounds or radicals allow important advances in defining biological activities and in structure-activity studies. Under this perspective, our research group designed and synthesized IR-01 (*Z*)-4-((1,5-dimethyl-3-oxo-2-phenyl-2,3dihydro-1*H*-pyrazol-4-yl) amino)-4-oxobut-2-enoic acid using 4-aminoantipyrine associated to the structural fragment 1,4-dioxo-2-butenyl, observing the influence of the position of the phenyl ring, the distance between fragments, the increased number of heteroatoms and the increased number of olefins, as indicated in the literature (Jha *et al.*, 2010).

Often, the synthesis of a biologically effective compound can use reagents and/or approaches of considerable environmental impact. Thus, compared with the efficient but environmently agressive synthetic procedure described in the literature (Cunha *et al.*, 2005), our one-pot method assisted by microwave irradiation can be a similarly efficient but cleaner approach for the synthesis of IR-01, with a very good yield and a reaction time of only 1% compared to that reported in the literature.

The results of the biological studies on IR-01, prepared using this new synthesis method, suggest that it can cause DNA damage. However, this damage does not become fixed in the cell genome, a hypothesis that is reinforced by the fact that the damage evaluated by the comet assay did not result in a significant increase in micronucleus frequency. According to Rundell *et al.* (2003), genotoxic damage is likely to undergo repair, whereas mutagenic damage is not, with changes becoming fixed in the genetic material as mutations. Such occurrence is not uncommon in preclinical chemoprevention experiments, both with natural products (Synder and Gillies, 2002; Cunha *et al.*, 2005; Rodeiro *et al.*, 2006; Hoshina *et al.*, 2013; Mendanha da Cunha *et al.*, 2013; Luo *et al.*, 2015) and with synthetic compounds (Zhan *et al.*, 2008; Cao *et al.*, 2015; Frolova *et al.*, 2015; de Araújo *et al.*, 2017). Although there are significant differences in the frequency of micronuclei between the negative control group and the groups treated with IR-01, Vaz *et al.* (2016) describes that this isolated fact does not necessarily imply in toxicogenetic damage. Also, according to the results observed in other experiments from our research group (Oliveira *et al.*, 2009a, 2013; Mauro *et al.*, 2010; Pesarini *et al.*, 2014; Navarro *et al.*, 2015), the baseline frequency of micronuclei can be greater than the frequency observed for the animals treated with IR-01 in the present study. For example, according to Oliveira *et al.* (2015b), the baseline frequency of micronuclei in Swiss female mice may reach 11.00 ± 3.16.

Based on these data, IR-01 is considered to cause genomic damage but is unable to cause chromosomal damage. The genotoxic activity may occur because of the pharmacophore 1,4-dioxo-2-butenyl, which has already been described as an effective cytotoxic agent in the tumor cell lines Molt4/C8 and CEM L1210 (Jha *et al.*, 2010). However, the addition of 4-aminoantipyrine may have modified this property, which is required in anticancer agents. It is important to highlight that chemotherapeutic agents generally include in their mechanism of action the induction of DNA damage that causes cell death, especially that of tumor cells (Elsendoorn *et al.*, 2001; Nadin *et al.*, 2005).

The chemopreventive action described may be explained by IR-01, which originated from 4-aminoantipyrine, retaining the antioxidant activity of its precursor. This hypothesis is consistent with the data from the present study because the best capacity for preventing genomic damage were observed for doxorubicin and cyclophosphamide, chemotherapeutic agents that are capable of generating free radicals that cause rather extensive genotoxic damage (Almeida *et al.*, 2005), triggering cell death and thereby exerting their anticancer action. The lack of a pattern in the chemopreventive response of the three studied commercial chemotherapeutic agents suggests that their mechanisms of action may interfere with the response to DNA damage in the presence of IR-01.

Cisplatin is also a chemotherapeutic agent that generates free radicals (Antunes and Bianchi, 2004), and this needs to be considered in its antitumor action. However, when this drug was combined with IR-01, the rate of DNA damage reduction increased, contrary to what was observed for doxorubicin and cyclophosphamide. Thus, despite the existence of the 4-aminoantipyrine radical, the antioxidant activity was undetectable, and this increase in DNA damage reduction could be attributed to the pharmacophore 1,4-dioxo-2-butenyl. However, further studies are needed to clarify this potentiation of the toxicogenic effects. A hypothesis for discussion is the reduction caused by cisplatin in the activity of antioxidant enzymes, such as superoxide dismutase, catalase, GSH peroxidase and GSH reductase (Hyppolito and Oliveira, 2005). Thus, the oxidative and consequently genotoxic capacity of cisplatin is more intense than that of the previously cited antineoplastic agents, which may have led to the increased genomic damage.

Regarding the micronucleus assay, the pattern of response to doxorubicin resembled that shown for the comet assay, i.e., the percent damage reduction decreased with higher dose, showing an inverse correlation. Note that over time the chemopreventive activity decreased, but the inversely proportional response pattern was maintained. This pattern was expected, and the decrease in the chemopreventive activity was perhaps due mainly to the metabolization and elimination of IR-01. Metabolization and secretion were also observed for doxorubicin because the capacity for DNA damage induction decreased.

For cisplatin, a high percentage of damage reduction was observed at 24 hours. However, the percentages decreased over the three time points. These data suggest that the increased DNA damage observed in the comet assay was not fixed into the genetic material because no chromosomal damage occurred. This finding, in turn, suggests that the repair mechanism was effective in preventing that genomic damage would be fixed as chromosomal damage.

For cyclophosphamide, increased toxicogenic activity was observed over the 72 hours of the study. It occurred at the intermediate test dose despite its efficient capacity to prevent genomic damage, with reduced chemopreventive activity at the other two doses.

In addition to the ability to reduce commercial chemotherapeutics effects, IR-01 also has a pharmacophoric radical in its structure, which could increase the antioxidant defenses in non-injured cells. This hypothesis is supported by Bianchi and Antunes (1999), Albertini and Ruiz (2001), Antunes and Bianchi (2004), and Oliveira *et al.* (2013), who reported that cells that have DNA lesions are deficient in antioxidant defenses. Therefore, these cells are more prone to suffer cytotoxic damage and undergo cell death more easily when exposed to certain cytotoxic and/or genotoxic agents. On the other hand, when normal cells with adequate antioxidant enzyme activity are in contact with another antioxidant agent, there is less of a chance that DNA damage is caused by free radicals.

The splenic phagocytosis test revealed that the same toxicogenetic doses also stimulated splenic phagocytosis. Thus, cells with DNA damage were efficiently removed from the bloodstream. Other studies have shonw that the spleen has the ability to remove tumor cells and/or DNA-damaged cells that are prone to carcinogenesis from the bloodstream (Cruvinel *et al.*, 2010; Navarro *et al.*, 2014; Carvalho *et al.*, 2015). In addition, splenic phagocytosis is also associated with the biomonitoring of blood cell viability, and it promotes the removal of senescent leukocytes, platelets and erythrocytes (Freitas *et al.*, 2009), as well as of apoptotic bodies and pathogens (Huysentruyt and Seyfried, 2010).

The splenic phagocytosis analysis also showed that all the chemotherapeutic agents were able to increase splenic activity, which was expected because these agents cause DNA damage and these damaged cells tend to be sequestered. When the agents were associated with IR-01, a decrease in phagocytosis occurred in all the experimental groups. This result suggests that the absence of cells with chromosomal damage did not stimulate the spleen to increase splenic phagocytosis.

Considering the alterations in both leukometry and splenic phagocytosis caused by IR-01, interestingly, it was observed that this molecule was able to increase the number of circulating lymphocytes and to cause a reduction in neutrophil numbers. These data suggest that the increase in phagocytic activity may be characterized by neutrophils exiting the blood and migrating into the spleen to sequester cells with DNA damage. Similar findings have been reported in other studies that also identified immunostimulatory compounds through this association (Lee *et al.*, 2003; Leung *et al.*, 2005; Ishii *et al.*, 2011; Sang *et al.*, 2013).

The combination of IR-01 with doxorubicin and cisplatin also showed an increase in the number of leukocytes and a reduction in the number of neutrophils. These findings corroborate the increase in splenic phagocytosis observed and discussed for the previous assay.

No variation in the frequency of blood cells was observed for cyclophosphamide. However, this does not contradict the splenic phagocytosis observed for the two lower doses. According to Oliveira *et al.* (2015a), splenic phagocytosis can occur efficiently even in the absence of a change in blood cell counts.

The present study also assessed cell death, given that unrepaired cells with DNA damage tend to disrupt the cell cycle and enter apoptosis (Zhou and Elledge, 2000). The results showed that IR-01 is capable of increasing the frequency of dead cells, from now on considered apoptotic cells, in the liver and kidneys. This is an important issue because IR-01 can stimulate splenic phagocytosis, increase the number of lymphocytes, and induce cell death, despite that it can cause genomic damage without causing chromosomal damage. Such important biological activities may be required in chemopreventive compounds (De Flora and Ferguson, 2005).

Nevertheless, although IR-01 is capable of increasing apoptosis when administered alone, in combination with doxorubicin, cisplatin and cyclophosphamide, IR-01 it generally reduced the frequency of apoptosis caused by these agents. The potentiation of apoptosis could be a good indicator of its adjuvant action in chemotherapy. However, such potentiation occurred only for doxorubicin. Therefore, the results do not encourage the use of IR-01 in combination with chemotherapeutic agents in anticancer therapy.

Given the above data, IR-01 has properties that render it sufficient to be classified as a chemopreventive agent, such as the inability of causing chromosomal damage, antigenotoxic potential, and the ability to alter leukometry, increase phagocytosis and induce cell death. These properties are possibly correlated with the 4-aminoantipyrine radical, an important antioxidant moiety present in the IR-01 molecule and described as having anti-inflammatory, analgesic, and antipyretic properties (Burdulene *et al.*, 1999; Turan-Zitouni *et al.*, 2001; Pisoschi and Pop, 2015).

According to Fedel-Miyasato *et al.* (2014) and Rocha *et al.* (2015) a good correlation exists between effective anti-inflammatory and immunostimulatory actions and chemopreventive effects. As reported in the literature, antioxidants that block carcinogenesis can exert their chemopreventive function via two different lines of organic defense: (I) by preventing the formation of free radicals that interact with and degrade DNA and (II) by intercepting existing or newly formed free radicals in cells, thereby causing a delay or inhibition of oxidation rates (Maxwell, 1995). The latter is correlated with the desmutagenic mode of action of substances reported as potential chemopreventive agents (Sato *et al.*, 1984; Ferrara *et al.*, 2000; Pesarini *et al.*, 2013; Navarro *et al.*, 2015).

If these lines of defense are still insufficient, the body may also facilitate the excretion of xenobiotics through detoxification enzymes, making them more water soluble and, thus, assisting their elimination by the kidneys (Cordon-Cardo *et al.*, 1989; Hooiveld *et al.*, 2001), while modulating the DNA repair system. This last line of defense is associated with bioantimutagenesis, in which enzymes are modulated by test compounds, thus favoring the correction and integrity of the genetic material, reducing the probability of developing cancer (Oliveira *et al.*, 2009b; Nakamura *et al.*, 1999; Di Giacomo *et al.*, 2014; Leite *et al.*, 2015).

All of the actions that are attributed to IR-01 discourage its use in combination with chemotherapeutic agents because of the maintenance of the antioxidant activity of its precursor, 4-aminoantipyrine. This use is discouraged because despite the fact that IR-01 can cause genomic damage, can increase splenic phagocytosis, lymphocyte number and the frequency of cell death, all these being properties required for chemotherapeutic agents, IR-01 can interfere negatively when associated with drugs already used extensively in anticancer therapy. Such interference prevents DNA damage and apoptosis, which are the main pathways for the elimination of tumor cells.

Thus, we consider that IR-01 is not indicated for use as an adjuvant in anticancer therapy in combination with doxorubicin, cisplatin or cyclophosphamide. In this case, the properties derived from 4-aminoantipyrine, even when in combination with the 1,4-dioxo-butenyl fragment, recognized as cytotoxic (Jha *et al.*, 2010), largely overrode the ability to induce cell death. Corroborating this, the study by Berno *et al.* (2016) states that 4-aminoantipyrine, a dipyrone metabolite, reduces DNA damage, apoptosis induction and phagocytosi when administered in combination with doxorubicin, cisplatin or cyclophosphamide.

The present study is the first to propose a new synthetic methodology to efficiently and cleanly produce IR-01 and the first to demonstrate the chemopreventive effects of this molecule. In addition, we contraindicate the use of IR-01 as an adjuvant in anticancer therapies in combination with doxorubicin, cisplatin and cyclophosphamide because of its ability to reduce important effects of these agents.
